# Framing Migrant Drownings in Australia: News Media Representations Through the Lens of Critical Discourse

**DOI:** 10.1002/hpja.70176

**Published:** 2026-03-23

**Authors:** Emma Derainne, Ryan Essex, Jagnoor Jagnoor

**Affiliations:** ^1^ The George Institute for Global Health, UNSW Sydney, Level 8, Health Translation Hub Randwick New South Wales Australia

**Keywords:** Australia, critical discourse analysis, cultural sensitivity, drowning, media representation, migrant, migration, public policy, structural inequality, water safety

## Abstract

**Introduction:**

Media reporting of migrant drowning deaths can serve multiple purposes, including advocacy, improving data, and supporting inclusive policy development. However, such drownings remain underexamined in both public discourse and academic research. This study investigates how migrants are portrayed in Australian newspaper coverage of drowning between 2020 and 2025, and how these portrayals shape public understanding, reinforce or challenge systemic inequities, and align with the equity goals of the Australian Water Safety Strategy 2030.

**Methods:**

A total of 82 articles from Australia's six highest‐readership newspapers were analysed using Critical Discourse Analysis guided by Mullet's General Analytical Framework, alongside Braun and Clarke's thematic analysis to identify patterns of power, ideology, and representation. Media language was manually coded, and keyword frequencies were tallied to explore how responsibility and risk are framed.

**Results:**

Coverage consistently portrayed migrants as at‐risk ‘newcomers’, with official voices represented by lifesaving bodies, councils, and aquatic educators, shaping responses. Drowning risk was often individualised, while structural determinants such as access to lessons or facilities were inconsistently reported. Parallel narratives positioned aquatic participation as a marker of ‘Australian’ identity, implicitly othering migrants. At the same time, some reports highlighted multilingual programs, subsidised lessons, and infrastructure investment, pointing to systemic interventions. These representations both reinforced individual responsibility and underscored structural inequities.

**Conclusions:**

Australian news media shape public understanding of drowning risk, but coverage tends to emphasise individual adaptation over structural causes. Greater consistency in reporting systemic barriers and prevention initiatives is needed to support equity‐oriented water safety strategies.

**So What?:**

Aligning media representation with the Australian Water Safety Strategy 2030 requires greater inclusion of migrant voices, consistent reporting of systemic barriers, and framing prevention in equity‐oriented terms. Collaboration between journalists and water safety agencies could help shift coverage from episodic tragedy to sustained public health communication.

## 1 Introduction

Drowning is a preventable yet critical global health issue, causing an estimated 300 000 fatalities each year [1]. Migrants are disproportionately vulnerable due to systemic inequities, including limited access to water safety education, unfamiliarity with aquatic environments, language barriers and marginalisation from public health infrastructure [2, 3]. However, migrants are not a homogeneous group; they include international students, tourists, working‐holiday makers, skilled migrants, and long‐term residents, each with distinct experiences and risk profiles.

Media reporting plays a pivotal role in shaping drowning prevention efforts. Coverage influences injury surveillance, advocacy, and policy development [4, 5, 6]. In contexts where comprehensive official statistics are lacking, news outlets can act as an informal surveillance system [7, 8]. At the same time, media narratives strongly influence how the public perceives drowning risk [9].

In Australia, this influence is particularly significant for migrant populations. News reports can raise awareness, shape discourse, and influence policy, yet they can also perpetuate misinformed narratives about risk and responsibility. Media framing determines who is visible, what prevention messages are conveyed, and how accountability is assigned [7, 10, 11]. When migrant voices are absent or misrepresented, prevention cues may lack cultural relevance, undermining public health efforts.

Australia's national drowning prevention plan, the *Australian Water Safety Strategy 2030*, identifies migrants and multicultural communities as priority populations, noting that one‐third of drowning deaths involve people born overseas [9]. The strategy sets a target of reducing drowning among these groups by 50% by 2030 and explicitly calls for engagement with multicultural media. Media coverage that highlights inclusivity and prevention could help achieve these goals; conversely, coverage that marginalises victims may reinforce harmful biases.

Language in media discourse is also a key instrument of power [12]. Classic media research demonstrates that mass media institutions act as gatekeepers, selecting which events are reported and whose perspectives are amplified. Through agenda‐setting and framing, the media not only reflect but also actively construct social realities [10, 13]. In the context of drowning, this means that journalistic choices about wording, emphasis, and source selection shape how the public understands risk and responsibility. These dynamics often operate through subtle strategies rather than overt bias, such as framing migrants as the ‘other’ [14]. This positioning casts native‐born Australians as responsible and virtuous, while migrants appear risky or culpable. Over time, such portrayals normalise migrant drownings as inevitable, individualised tragedies rather than preventable public health issues requiring systemic action [15, 16].

Given this influence, it is essential to examine how drowning incidents involving migrants are reported in Australia. This study applies Critical Discourse Analysis [17] to print and online news coverage from 2020 to 2025 to identify bias, stereotyping, and differential reporting.

The discourses of interest were the portrayals of drowning involving migrant populations in Australian newspaper reporting between 2020 and 2025. The analysis was guided by the following questions:

1. How are drowning incidents involving migrants portrayed in Australian newspaper media from 2020 to 2025?
2. How do these portrayals compare with representations of non‐migrant drowning incidents over the same period?

By addressing these questions, this study seeks to clarify the media's role in shaping discourse on migrant drownings and to inform strategies for promoting inclusive, evidence‐based water safety messaging.

## 2 Methods

This study employed the General Analytical Framework for Critical Discourse Analysis (CDA) as outlined by Mullet [18], which consists of seven systematic stages. The discourse of interest was the media representation of migrant and non‐migrant drowning incidents in Australia between 2020 and 2025.

The analysis used the General Analytical Framework for CDA [18]. The General Analytical Framework systematically examines texts through seven stages, outlined in Table 1.

**TABLE 1:** Analytical Framework for CDA as applied for this analysis.

Stage of analysis	Description	Implementation in the context
Select the discourse	Select the discourse related to injustice or inequality in society	Media representation of migrants and non‐migrants in Australian news media from 2020 to 2025
2 Locate and prepare the data sources	Select data sources (texts) and prepare the data for analysis	156 newspaper articles from the 6 Australian newspapers with the highest readership were selected through a process of inclusion/exclusion criteria.
3 Explore the background of each text	Examine the social and historical context and producers of the texts	An analysis of the Australia media landscape was conducted to understand the Australian media discourse constructed through historical and social contexts.
4 Code texts and identify overarching themes	Identify the major themes and subthemes using choice of qualitative coding method.	An inductive thematic analysis was conducted using the Braun and Clarke method [19].
5 Analyse the external relations in the texts (interdiscursivity)	Examine the social relations that control the production of the text; examine the reciprocal relations and how these influence social practices and structures and, simultaneously, how those constructions inform the arguments in the text as a continuing cycle of co‐construction of meaning.	Dominant social practices and norms (e.g., migrants as drowning victims), social structures (e.g., access to infrastructure, time in country, government, and institutional initiatives).
6 Analyse the internal relations in the text	Examined the language for indications of the aims of the texts (what is text trying to do), representation (e.g., representation of the social context, events, stakeholders), and the writer's positionality	Examined the headlines and the leading statements. Which voices are foregrounded or backgrounded; who is being quoted and why? what sensitised vocabulary is being used?
7 Interpret the data	Interpret the meanings of the major themes, external relations, and internal relations identified in stages 4, 5, 6.	Analysed structural features and individual fragments and situating them in the broader context of drowning prevention and themes established in the earlier stages.

In Stage 2, data sources were identified in the ProQuest database using the terms ‘drown*’ and ‘Australia’ (title/abstract/full text). The focus was on the six newspapers with the highest readership across print and digital platforms as outlined in Table 2.

**TABLE 2:** Readership of Top Australian Newspapers for the calendar year 2024.

Newspaper	Paper readership	Online readership	Paper + online readership
Sydney Morning Herald	820 000	4 125 000	4 590 000
The Age	690 000	2 810 000	3 180 000
Herald Sun	1 050 000	2 015 000	2 840 000
Daily Telegraph	1 095 000	1 920 000	2 735 000
The Australian	820 000	1 150 000	1,820 000
Courier‐Mail	830 000	1 150 000	1,820 000

*Note:* Adapted from Roy Morgan (2024).

### 2.1 Inclusion

- Published between 2020 and 2025 in one of the six highest‐readership Australian newspapers [20]: Sydney Morning Herald, The Age, Herald Sun, Daily Telegraph, The Australian, and Courier Mail.
- Focused on drowning incidents within Australia (beaches, rivers, pools, lakes)
- Identified victims as migrants (M) (e.g., international students, tourists, temporary/permanent residents) or non‐migrants (NM) (Australian‐born or naturalised citizens) or both migrants and non‐migrants (B) or neutral (N).
- Full‐text available in English via ProQuest.

### 2.2 Exclusion

- Articles not about drowning (e.g., metaphorical use), involving overseas incidents, outside the date range, or referring to migrants in transit.
- Duplicates, letters to the editor, editorials, opinion pieces, lifestyle columns, or other non‐news formats

**FIGURE 1:**
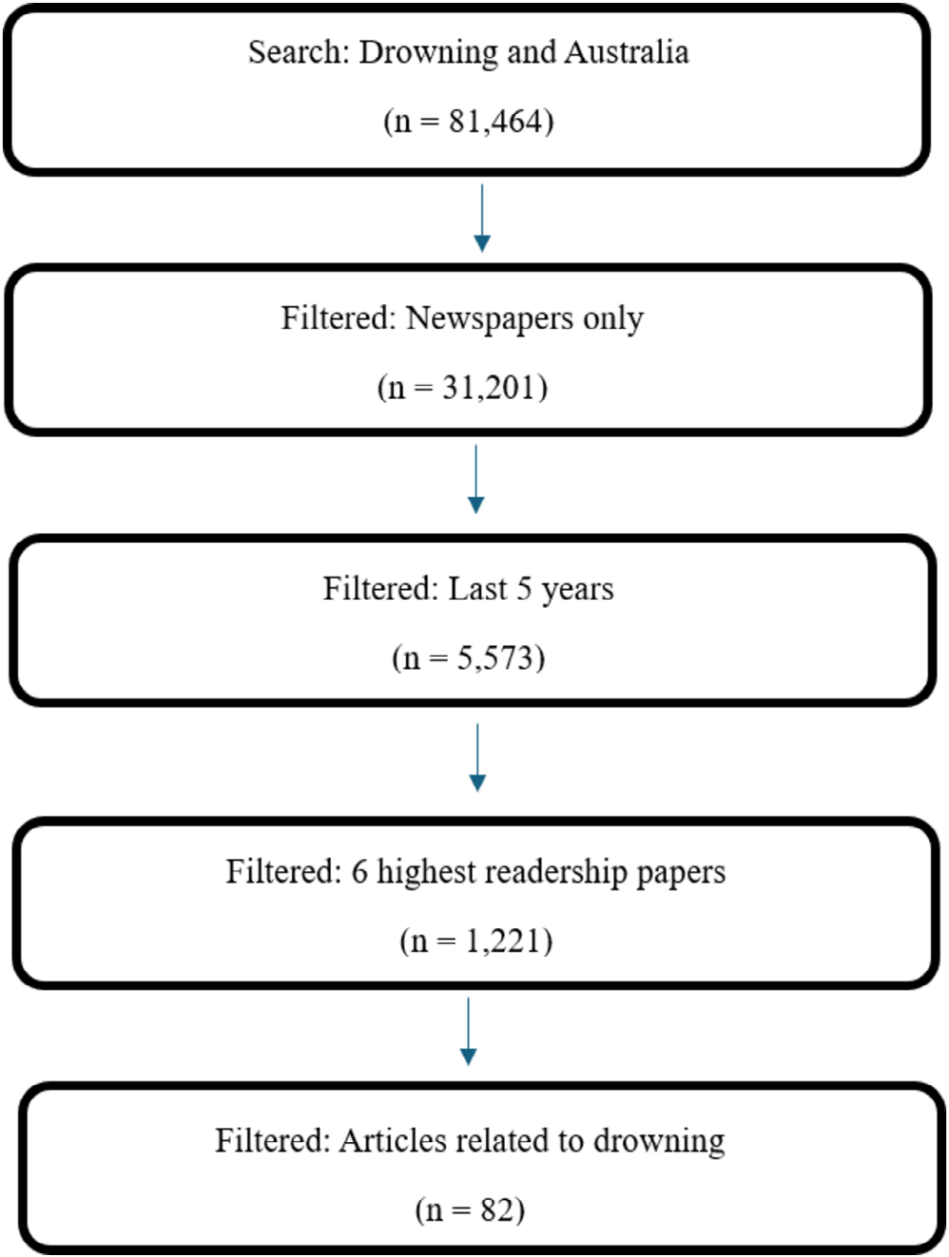
Flow chart of search strategy.

The search initially returned 81 464 results; of those, 82 were retained based on relevance to migrant drowning in Australia as outlined in Figure 1. Given the widespread syndication of news content across Australian mastheads, we defined duplicates as articles that were identical or near‐identical in narrative content (e.g., same event, but also same quoted sources and same phrasing), even if published in different newspapers or with minor headline edits. Where near‐identical versions appeared across multiple mastheads (e.g., The Age and Sydney Morning Herald), we retained a single instance to avoid over‐weighting syndicated reporting. Articles were treated as distinct (and retained separately) when they contained meaningful differences in framing, sources quoted, prevention messaging, or contextual details, even if they referred to the same drowning incident. Duplicate screening was conducted manually by comparing headline, publication date, named victim/event details, and overlapping verbatim text.

Stage 3 involved contextual mapping of the Australian newspaper landscape to inform interpretation of discursive patterns. We documented (i) ownership concentration and cross‐publication syndication across included mastheads; (ii) editorial orientation and audience positioning (e.g., tabloid vs. broadsheet, metropolitan vs. state‐based) and (iii) routine sourcing practices in drowning coverage (e.g., reliance on police, surf lifesaving bodies, councils, and coronial information). These contextual factors were used as an interpretive lens during Stage 6–7 to assess whose voices were routinely privileged, how migrant communities were positioned within dominant ‘risk’ narratives, and how structural explanations were foregrounded or backgrounded. This ensured that patterns in voice and framing were not treated as purely textual features, but as products of an ownership‐ and institutionally shaped media environment. Notably, six mastheads are concentrated within two dominant publishers, which also increases the likelihood of syndication and shared editorial norms; the context was considered during interpretation of recurring frames and source patterns.

Stage 4 identified overarching themes and generated keyword‐frequency outputs. Prior to analysis, texts were cleaned to remove duplicate articles, headline captions, and non‐editorial content. Common stop words (e.g., the, and, they), punctuation and numerals were excluded. Words directly related to the search strategy (e.g., drown, drowning) were also removed to avoid circularity.

Keyword frequencies were calculated following manual coding of salient lexical items identified through iterative close reading. Keywords included both single terms and meaningful bigram (e.g., international student), consistent with discourse‐analytic approaches that privilege semantic relevance over purely automated outputs. Frequencies were normalised per 1000 words to allow comparison between migrant‐focused and non‐migrant articles of differing lengths. Normalised frequencies informed the comparative bar charts, while raw counts were retained to illustrate absolute prominence.

Keyword selection was guided by critical discourse analysis principles, focusing on evaluative, affective and attributional language rather than exhaustive lexical coverage. This approach prioritises discursive function over sentiment polarity and is consistent with qualitative media analysis, where meaning is contextually produced rather than algorithmically inferred. Quantitative outputs were therefore interpreted alongside inductively generated themes as opposed to standalone measures using Braun and Clarke [19].

Stage 5 explored interdiscursivity by situating the themes within broader social practices, examining how media portrayals of drowning reflect and reproduce dominant norms. ‘Othering’ was used as an analytic lens to understand how subordinate groups are discursively constructed [21, 22].

Stage 6 examined linguistic devices, including grammar, tone, rhetorical moves, metaphor and voice foregrounding/backgrounding, drawing on Huckin's [7] account of how textual prominence and silences shape meaning.

Stage 7 synthesised themes and linguistic patterns across the analysis and interpreted these in relation to the wider context of drowning prevention in Australia.

## 3 Results

The findings compare newspaper reports using the keywords’ Australia’ and ‘Drown*’ published between January 2020 and April 2025. Applying the inclusion and exclusion criteria outlined above resulted in 82 articles drawn from Australia's six highest‐readership newspapers Sydney Morning Herald (*n* = 24; 29.3%), The Age (*n* = 5; 6.1%), Herald Sun (*n* = 14; 17.1%), Daily Telegraph (*n* = 16; 19.5%), The Australian (*n* = 7; 8.5%) and Courier‐Mail (*n* = 6; 7.3%). Of these, seven articles focused primarily on migrants, while a further 15 articles included reference to both migrants and non‐migrants. The Sydney Morning Herald published the most migrant‐focused articles (*n* = 8). The remaining articles addressed drowning in more general terms without specific reference to migrant populations. Using a thematic analysis approach, we inductively identified five main discourse strands: (1) Media language, (2) Migrant Drowning Death Reporting Patterns. (3) Emphasising migrant vulnerability, (4) constructing otherness and (5) prioritising either structural or individual responsibility. In this section, discourse strands are presented and discussed. We also observed two areas where these discourse strands entangled with each other.

### 3.1 Media Language

**FIGURE 2:**
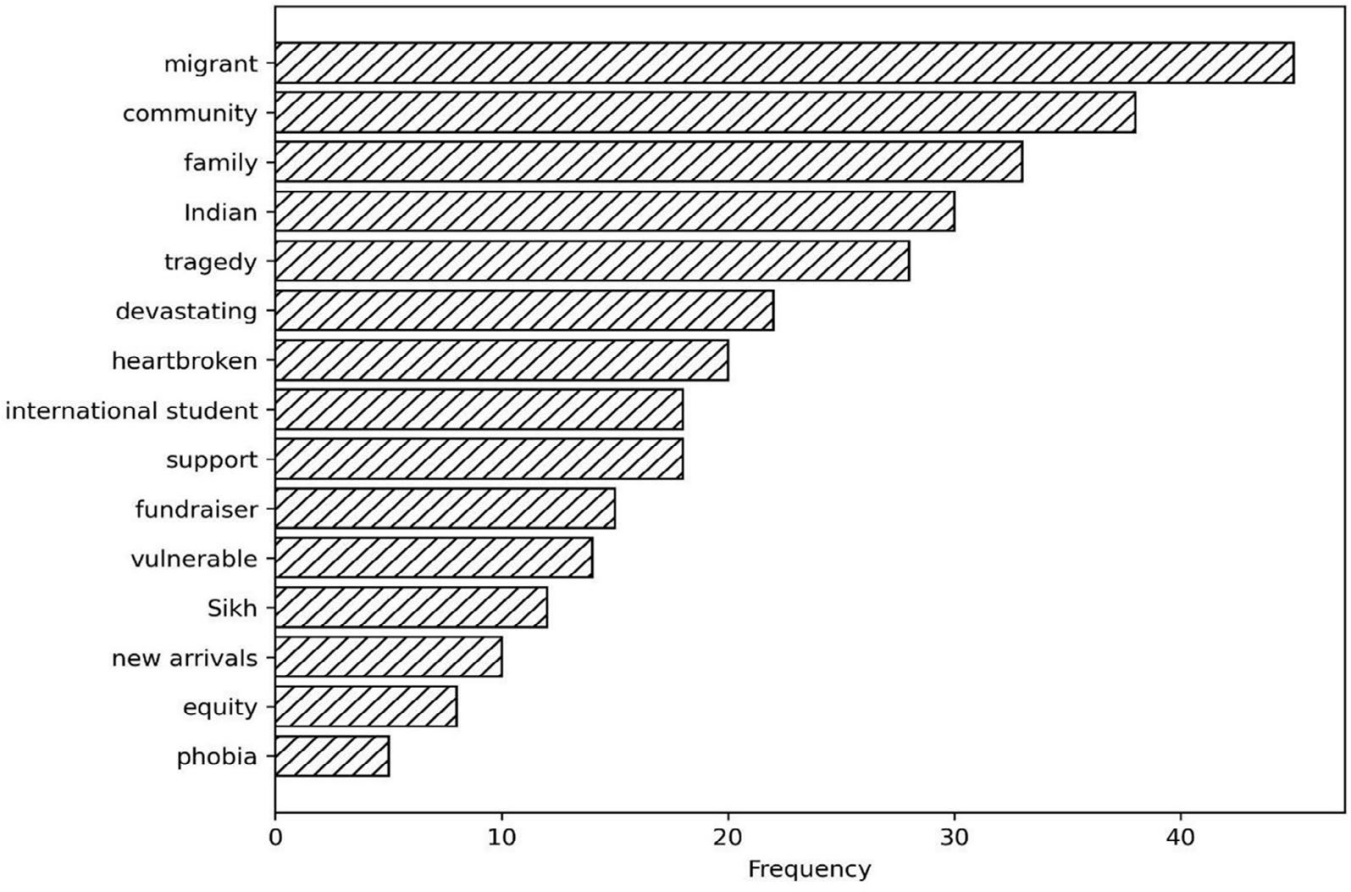
Frequency of keywords in Migrant Reporting.

**FIGURE 3:**
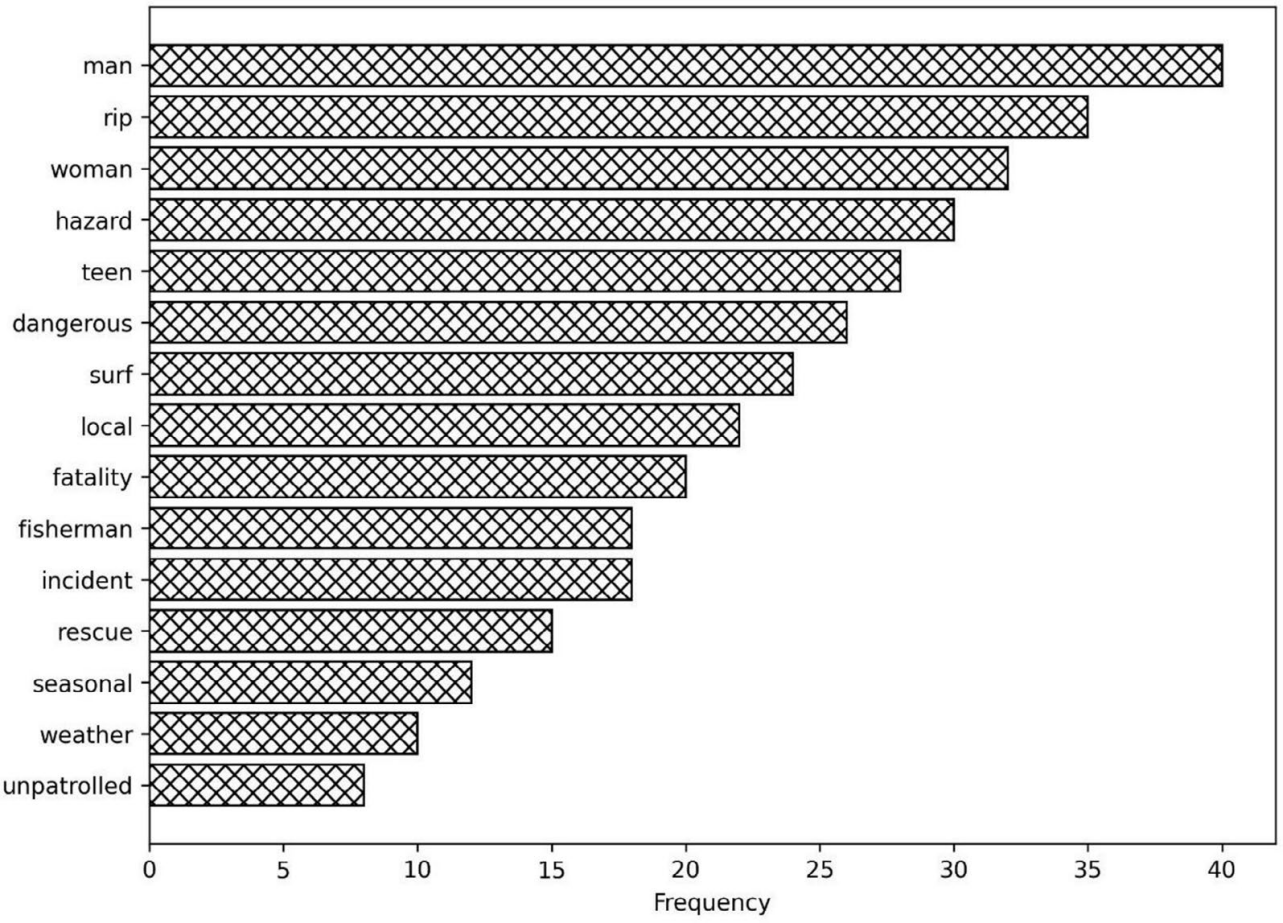
Frequency of key words in Non‐Migrant Reporting.

Migrant drowning reports consistently use personalised and emotive framing (kinship, aspirations, cultural identity, community grief), while non‐migrant drowning reports lean towards neutral, depersonalised and environmental framing (hazards, conditions, statistics) as outlined in Table 3. Figures 2 and 3 outline the word frequency in migrant and non‐migrant reporting.

**TABLE 3:** Key word comparison between migrant reports and non‐migrant reports.

Category	Migrant reports	Non‐migrant reports
Victim Identity	Indian‐Australian, Congolese, international student, migrant family, Indian national	Man, woman, teenager, fisherman, swimmer
Personalisation	Beloved son, golden‐hearted boy, happy‐go‐lucky father, hardworking student, visiting family	58‐year‐old fisherman, 66‐year‐old woman, 14‐year‐old boy
Emotive Language	Tragedy, devastating, heartbroken, haunts, traumatised, community reeling	Unfortunate, incident, fatality, pronounced dead, accident
Cause/Attribution	Unfamiliar with conditions, dangerous rip, not trained, no swimming lessons, migrant vulnerability	Hazardous surf, dangerous coastline, rip currents, environmental conditions
Community Framing	Mourning community, fundraiser launched, Sikh temple prayers, support for family	Calls for caution, warnings issued, renewed safety campaigns
Systemic/Equity References	Lack of swimming lessons, multicultural outreach, new migrants, barriers to access, equity gap	Patrolled vs. unpatrolled beaches, safety signage, weather alerts

Analysis of drowning reports reveals a clear divergence in media language between migrant and non‐migrant cases. Migrant reports consistently used personalised and emotive framings, describing victims through kinship and affective labels (e.g., beloved son, golden‐hearted boy) and portraying deaths as tragedies that left communities’ reeling’. In contrast, non‐migrant reports adopted neutral, depersonalised and environmental framings, referring to victims by generic descriptors (e.g., man, woman, fisherman) and attributing causes to hazards such as rips, surf or weather. Quantitatively, migrant reports contained 72% of emotive terms and over 90% of kinship references, while non‐migrant reports contained 69% of structural/hazard terms and 77% of solution‐oriented language. In this analysis, emotive terms refer to words and phrases that explicitly convey affect or moral evaluation (e.g., tragic, heartbroken, devastating, beloved), which function discursively to personalise loss and elicit empathy; an established linguistic strategy for heightening news value and shaping audience identification [23].

Migrant reporting also foregrounded inequities such as lack of swimming lessons and barriers to access, whereas non‐migrant reports emphasised general safety infrastructure. These patterns suggest migrant drownings are disproportionately framed through personal deficit and community grief, while non‐migrant drownings are situated within structural risks and technical solutions, reinforcing migrant ‘otherness’ and obscuring systemic determinants of risk.

### 3.2 Migrant Drowning Death Reporting Patterns

Of the 82 drowning reports analysed, 22 (27%) referenced migrants in some way. This proportion is lower than the proportion of drowning deaths involving people born overseas reported in national drowning statistics (~one‐third), suggesting migrants are not proportionately visible across drowning reporting overall. However, when migrant drownings were covered, they were more likely to receive intensive, clustered coverage: multiple follow‐up stories about the same incidents, substantially longer article length and greater use of personalised and emotive narrative elements. In contrast, many non‐migrant drowning deaths were reported as short episodic items or seasonal warnings, with fewer extended narratives. This indicates an asymmetry not simply in volume, but in the news values and storytelling style applied to migrant versus non‐migrant deaths.

### 3.3 Emphasising Migrant Vulnerability

A consistent theme across reporting was that migrants were frequently identified as vulnerable to drowning, often linked to cultural or linguistic background and sometimes compounded by socioeconomic status and age. One Sydney Morning Herald (19 March, 2025) article noted that water safety initiatives ‘had not reached those at greatest risk of drowning, including children from lower socioeconomic families, and/or culturally and linguistically diverse communities, and it ignored adults’.

Vulnerability was framed through both cultural background and socioeconomic position, linking drowning risk to demographic identity rather than broader structural determinants. Media outlets often positioned migrants as the primary target of preventative efforts. For example, following a cluster of summer drownings in Queensland, the Courier‐Mail (January 2024) reported that ‘authorities urged migrant families to take up lessons and avoid unpatrolled beaches after a series of incidents’ while the Daily Telegraph (28 December, 2023) quoted experts who ‘pleaded for weak swimmers, including children and those of migrant backgrounds who may have less swimming experience, to be careful or just stay out of the water’. A Surf Life Saving spokesperson added that ‘everyone enjoying Australia's waterways needed to prioritise safety, especially migrants who may not have had the same opportunities to learn water‐safety skills in their youth’ (The Australian, 28 December, 2022).

Migrant overrepresentation was reinforced through statistics and repeated reporting. Migrants were repeatedly identified as an ‘at‐risk’ group, with reporting frequently citing national statistics to underline this risk: ‘half of all beach drowning deaths in Australia since 2004 have been people born overseas’ (The Daily Telegraph, 28 December, 2023). Other coverage framed risk through access and prevention reach, noting that initiatives ‘had not reached those at greatest risk…including…culturally and linguistically diverse communities’ (Sydney Morning Herald, 19 March, 2025). Some articles acknowledged heterogeneity within migrant populations, noting that vulnerability varied depending on ‘how old they are when they arrive, what their perception of coastal safety is’ (The Daily Telegraph, 13 September 2024). A smaller counter‐narrative highlighted that ‘Migrant groups were doing great work in informing communities about water safety’ (The Daily Telegraph, 13 September 2024).

When reporting specific drowning incidents, the media frequently foregrounded migrant or tourist identity, often highlighting nationality or cultural background. For example, one report described ‘a 43‐year‐old woman visiting Australia from India’ (Herald Sun, 13 September, 2024); another noted ‘a Brazilian man believed to be a holidaymaker’ (The Daily Telegraph, 27 January, 2023), and a family tragedy described ‘a Nepalese father of two who drowned at Glenelg beach after entering the water to rescue his children’ (Courier‐Mail, February 2023). By contrast, non‐migrant drownings emphasised context and environment rather than cultural identity. For instance, the Courier‐Mail reported that ‘Sonya Knyf, 51, was swimming with her 14‐year‐old daughter…when they ran into difficulty in the rough condition. Lifesavers and good Samaritans…ran to their aid, but Mrs Knyf died at the scene’ (2 May, 2023), and the Herald Sun described ‘the deaths of a 17‐year‐old boy at Mordialloc and a man in his 30s at Ebden as reminders of the real dangers that can quickly trap the unwary or inexperienced’ (December 2021). Another Herald Sun report emphasised multiple families caught in misfortune: ‘three families are left in mourning in the wake of drownings as summer beats down in Victoria and thousands flock to the water’ (December 2021).

Climate and extreme weather were rarely linked to migrant drowning narratives. While some non‐migrant reporting referenced heat, flooding, storms or extreme conditions as contextual contributors to risk, migrant‐focused articles predominantly framed risk through unfamiliarity, swimming ability or behavioural adaptation, with minimal reference to climate‐related drivers.

Overall, migrant drownings were more likely to be described with reference to ethnicity, nationality or migrant background, while non‐migrant cases emphasised environmental dangers or situational misfortune. This contrast illustrates how migrants may be constructed as a distinct or homogenous group in media narratives, with potential implications for public understanding and the equity goals of the Australian Water Safety Strategy 2030.

### 3.4 Constructing Otherness

The discourse of otherness appeared most clearly when reporting on swimming ability and its relation to Australian national identity. Migrants were often positioned as outsiders to a national culture built around water, constructed not as individuals with varied experiences but as a collective marked by absence of skills, familiarity, or belonging. Swimming was framed simultaneously as a safety practice and as a civic marker of membership. News items drew on historical memory and nostalgia to reinforce this ideal: ‘Compulsory swimming lessons were introduced to NSW schools in the 1880s as part of a nation‐building program that came to define what it was to be Australian’ (Sydney Morning Herald, 19 March, 2025).

Contemporary reporting also highlights population‐wide deficits in swimming ability (e.g., one in four adults is a weak or non‐swimmer, and many children leaving primary school are unable to swim), suggesting that attributing risk primarily to migrant identity may obscure a broader, national vulnerability. This was paired with concern that the tradition is eroding, as another piece lamented that ‘the Australian rite of passage in which every school student swam or participated in the annual swimming carnival is dying’ (Sydney Morning Herald, 18 March, 2025). Lifestyle narratives extended this idea from skill to identity, normalising beachgoing as a core cultural practice: ‘Swimming and outdoor lifestyle, like going to the beach, is central to who we are’ (Sydney Morning Herald, 19 March, 2025).

Swimming thus operated rhetorically as both leisure and citizenship, implying that to be Australian was to be naturally competent in the water. Migrants were contrasted as ‘new arrivals’ lacking knowledge of beaches or surf. However, such contrasts risk overstating cultural difference, as recent national data indicates that swimming ability is declining across the Australian population. Reports describing Australia as a ‘nation of waders’ (SBS, 2025) highlight that many adults and children, regardless of background, now lack the swimming proficiency once associated with national identity.

Reports regularly contrasted visitors and migrants with ‘locals’ by framing newcomers as unaware of hazards or ‘not knowing the water’. One typical explanation emphasised the ease with which an inexperienced visitor might enter unsafe conditions: ‘When somebody comes down just for a day and really doesn't know the area, they see a car park and a beach and it looks nice, they just go in …. For people who haven't grown up around beaches, they might not know the danger of the surf’ (The Age, 27 January, 2024).

This portrayal often locates risk in cultural unfamiliarity rather than in systemic issues such as insufficient multilingual signage. ‘New arrival’ became shorthand for risk, flattening diverse experiences into a simplified narrative of inexperience: ‘Many new arrivals to our country haven't previously heard those warnings or otherwise imagine that our beaches are as mild as beaches elsewhere. That's when the risk of drowning can turn very real’ (The Daily Telegraph, 28 December, 2023).

Here, ‘new arrivals’ becomes a catch‐all category, flattening diverse experiences into a simplified narrative of inexperience. The rhetorical move is significant because it ties risk to cultural difference, with limited acknowledgement of resilience, adaptation, or prior water safety knowledge among migrant populations. By contrast, non‐migrant drownings were rarely referenced cultural identity. These deaths were narrated through neutral descriptors (‘mother’, ‘teen’, ‘local’) and framed as products of environmental risk (dangerous surf, seasonal hazards), making non‐migrant identity the unmarked norm.

Alongside this dominant othering narrative, a smaller but consistent inclusionary strand highlighted participation pathways through junior surf lifesaving programs. Nippers was presented as an entry point where migrant families could belong and contribute. ‘Nippers has been an institution on our beaches for a century and now has young people from more than 80 different nationalities’ (Sydney Morning Herald, 2 March, 2024). Nippers was one of the few instances where migrants were consistently spoken about positively in relation to water safety and drowning prevention. Coverage framed Nippers as a counterpoint to deficit discourses: a site of belonging, participation, and shared practice. This participation frame signalled an expanding imaginary of the national community organised around shared practices rather than origin [13] (Hooks 1990). ‘Nippers is a rare kids' sport where there is no standing on the sidelines watching—the whole family can get involved,’ Burrows said. (The Age, April 2024). Here, migrants were framed not only as learners but as contributors to the cultural fabric of beach life. Nippers became a discursive counterweight to deficit narratives, representing migrants as participants in a shared tradition rather than perpetual outsiders. More importantly, Nippers was consistently depicted as a counterpoint to deficit discourses, portraying migrants as learners and contributors to a shared tradition. Coverage emphasised its family‐based model, suggesting that parents and children could learn together and overcome barriers such as cost and access.

Although a systematic temporal analysis was not undertaken, the reporting did include occasional counter‐discourses alongside dominant narratives of otherness. Coverage of the Nippers program, while less common, provided a notable example. These stories departed from deficit framings and instead highlighted the heterogeneity within migrant populations ‘Our Nippers come from backgrounds and countries all over the world, including youngsters from Kazakhstan, Bulgaria and South Korea,’ Surf Life Saving Australia's (SLSA) general manager Donna Wishart said. (Daily Telegraph, January 2025). By naming specific groups, reporting sought to make multicultural participation tangible rather than abstract, shifting the narrative from absence to contribution.

The comparative pattern is clear. While many articles drew on deficit framings that highlighted cultural unfamiliarity, ‘new arrival’ status, or lack of adaptation to Australian conditions, others offered inclusive counter‐narratives that emphasised migrant contribution through family participation and community cohesion. These accounts may indicate that programs such as Nippers are beginning to shift the narrative, with family‐based participation potentially creating opportunities for migrants to feel integral to, and actively engaged in, Australia's culture of water safety. In this framing, cultural influence and shared practice could support migrant inclusion and, over time, may contribute to a more plural and participatory understanding of national identity in relation to water safety.

### 3.5 Prioritising Either Structural or Individual Responsibility

The discourse framing of migrant drowning in Australian news media appeared to oscillate between two competing perspectives. The first highlighted structural factors, pointing to systemic barriers such as limited access to swimming lessons, inadequate infrastructure, or insufficient government investment in consistent water safety programs that may disproportionately affect migrant populations. The second framed drowning primarily as an individual issue, attributing migrant deaths to personal behaviours, cultural unfamiliarity or failure to adopt water safety practices, and suggesting individual‐level solutions.

Within the water safety discourse, the government and surf rescue organisations (Royal Life Saving Society of Australia and Surf Life Saving Australia) were sometimes portrayed as ‘duty bearers’, responsible for ensuring the protection of individuals and communities through policies, strategies, and prevention programs, while individuals and communities are described as the ‘right‐holders’ entitled to equitable access to safety measures and education. This dual positioning suggested that drowning prevention was both a structural obligation and a matter of personal adaptation. Some reports did acknowledge structural costs, noting that swimming lessons were increasingly unaffordable for many families and that the cost‐of‐living pressures and school budget restrictions limited access to formal swimming and water safety education. These financial barriers were occasionally linked to migrant and lower socioeconomic households, suggesting that economic inequity compounded other barriers to participation. However, similar barriers were also evident for low‐income Australian families, particularly in outer‐urban and regional areas such as Western Sydney, where infrastructure shortages and long travel distances limited access to public pools and lessons. Framing affordability and access primarily as a ‘migrant’ issue, therefore risks occulting broader structural inequities that apply across the diverse Australian population. Media reports often blurred these boundaries, with migrants more frequently framed through discourses of individual responsibility even when structural barriers were acknowledged.

The structural approach was also evident in coverage that discussed access, infrastructure and systemic design. Several articles pointed to inequities in the distribution and quality of facilities. ‘More than 6 million Australians are more than a 10‐minute drive from a public pool, 1.8 million are more than 20 minutes away….Blacktown has five pools for 435,000 people – one for every 80,000 residents’ (Sydney Morning Herald, 5 March, 2025). Here, risk was constructed as a matter of planning and investment, where geographic inequities shape who has the chance to learn to swim.

Only occasionally were migrant voices included as part of structural solutions. One report noted a Sikh‐led organisation collaborating with Congolese families, while Surf Life Saving highlighted junior programs as fostering cohesion. These accounts framed migrants not as passive recipients but as active agents. Yet they were presented as isolated vignettes rather than sustained narratives. ‘Harpreet has a phobia of water, but he knows his community needs help. His Sikh Volunteers Australia organisation has been working with the Congolese community and others to address gaps in swimming skills’. Elsewhere, Surf Life Saving Australia framed its junior programs as a bridge for migrant inclusion. ‘(the club) is really inclusive… bringing people in from all of these different backgrounds… helps with that community cohesion’ (The Daily Telegraph, 24 January, 2025).

Such examples demonstrate the potential of migrant‐led or inclusive approaches to reframe drowning prevention, but their rarity underscores how structural solutions were marginalised in favour of individual responsibility. Alongside this structural discourse, individual responsibility was a dominant frame across reporting. Coverage frequently placed responsibility on swimmers to evaluate their own behaviour and preparedness. As one official insisted: ‘we'll always advocate for more swimming lessons in the community, but we just really want to see people taking responsibility and risk‐assessing their behaviour around water’ (The Australian, 28 December, 2022). Here, drowning prevention was framed less as a systemic obligation and more as a matter of personal vigilance, with risk management pushed onto the individual swimmer. Importantly, this framing was not limited to any one group: the emphasis on personal responsibility and adherence to safety slogans such as ‘swim between the flags’ applied broadly to all beachgoers, irrespective of migrant status. However, when migrants were the focus on reporting, these universal messages were often accompanied by additional cultural or behavioural explanations, which subtly reinforced perceptions of difference.

Migrants featured prominently within these individual responsibility discourses. Reports drew attention to statistical overrepresentation noting that ‘Australian migrant communities were over‐represented in drowning cases, with only 15 per cent of migrants aged 35 and over having had formal swimming training’ (The Daily Telegraph, 24 January, 2025). This framing foregrounded migrant deficit, constructed as lack of training, while backgrounding structural constraints such as cost, accessibility, and cultural barriers, which are not evenly distributed across migrant individuals.

Prevention advice was explicitly directed at migrant families, urging them to avoid risk: ‘authorities urged migrant families to take up lessons and avoid unpatrolled beaches after a series of incidents’ (Courier‐Mail, January 2025). Advice targeted in this way both acknowledged migrant vulnerability and reinforced their positioning as a group requiring special instruction, further entrenching the assumption that cultural adaptation was the key solution. At times, the framing suggested failure to prepare for Australian conditions, such as the widely reported case of ‘Canadian grandfather Ron Brean, 79, who had been in Australia for only five days…went for an afternoon swim only to be swept into a fatal current’ (Daily Telegraph, 28 December, 2023). By foregrounding his newcomer status, the article subtly linked risk to inexperience with Australian waters rather than to the systemic absence of signage, patrols or contextual warnings.

Even when acknowledging migrant ‘deficits’, reporting tended to naturalise vulnerability as cultural or personal responsibility, while obscuring systemic barriers such as cost, access, and inclusion. Framing the issue in this way risks leaving the impression that personal adaptation is sufficient, irrespective of broader public health or resource shortcomings. While individual vigilance is undeniably important, structural conditions fundamentally shape people's opportunity to act safely—or fail to. Children from culturally and linguistically diverse backgrounds were positioned as needing structured inclusion: ‘(the club) wants to involve kids who may not necessarily be comfortable around water or the beach… potentially lifesaving skills are being acquired along the way’ (The Daily Telegraph, 24 January, 2025). This framing reinforced an assumption of deficit while also recognising the potential of programs like Nippers to bridge gaps.

References to structural factors, including pool access, patrol coverage, funding shortfalls, national strategies, appeared regularly but were typically relegated to background paragraphs or expert sidebars. By contrast, when migrants were the subject of an incident story, the dominant register was instructional and individualised. Headlines and articles foregrounded migrant identity (‘new arrivals’, tourists, international students) and paired it with behavioural injunctions (‘take lessons’, ‘avoid unpatrolled beaches’, ‘swim between the flags’). Comparable incidents involving long‐term residents were narrated through environmental hazards or seasonal risks, highlighting asymmetry in how responsibility was assigned.

Migrant voices themselves were sparsely present, with experts and agencies speaking ‘about’ rather than ‘with’ communities. When migrant‐led initiatives were mentioned, they appeared as isolated vignettes rather than a sustained counter‐frame. This indicates a dual discourse. At the all‐of‐system level, structural barriers such as inadequate infrastructure, unequal access to lessons, and gaps in government investment were occasionally acknowledged, yet rarely foregrounded as central causes. At the all‐of‐society level, cultural norms around swimming and national identity reinforced the assumption that migrants were less familiar or less competent in aquatic settings. Within this framing, migrant drowning was more often narrated through individual responsibility and identity‐based deficit, whereas non‐migrant drowning was narrated through context and hazard. The net effect is a bifurcated framing, in which similar risks were constructed as matters of personal adaptation for migrants but situational misfortune for non‐migrants.

## 4 Discussion

Australian media reporting of drowning presents both limitations and opportunities in how migrant experiences are represented. Interpreting these patterns within the contemporary Australian media landscape matters. The sample represents a highly concentrated news environment, where a small number of publishers shape a large proportion of what the public reads and shares. In practice, this concentration amplifies recurring sourcing routines, for example, police and surf lifesaving spokespeople, and can normalise particular framings of risk and responsibility. This helps explain why migrant drownings are repeatedly narrated through ‘new arrival’ and ‘lack of familiarity’ storylines, while structural determinants appear more intermittently or in backgrounded paragraphs. In other words, the findings are not simply about individual journalists’ word choices; they reflect the institutional conditions of reporting, including ownership concentration, syndication, and reliance on official sources.

While cultural‐difference explanations remain common, coverage also includes moments of visibility, solidarity and prevention messaging that can support public health efforts. This analysis shows how media can contribute to equity‐oriented prevention and align with the Australian Water Safety Strategy 2030 [24]. At the same time, significant gaps remain. Only 22 of 82 articles referenced migrants at all, highlighting how agenda‐setting influences whose experiences are considered newsworthy. The limited presence of migrant perspectives constrains prevention messaging and can reinforce inequities.

### 4.1 Responsibilisation

Australian media reporting largely individualises drowning risk. Migrant drownings were commonly attributed to personal behaviour, limited lessons, unfamiliarity, or poor judgement, while structural factors such as access, funding, or patrol availability received little attention. This reflects broader responsibilisation discourses [25], where individuals are tasked with adapting (‘swim between the flags’) regardless of structural barriers. Similar patterns can be found in other health domains. In the United States, obesity was framed as parental choice rather than food environments [26]; smoking as youth behaviour rather than a consequence of tobacco industry practices [27] and disaster deaths as evidence of ‘risk‐taking’ rather than systemic vulnerability [28]. During COVID‐19, migrants and minorities were similarly blamed for spreading the virus despite structural inequities [29]. Across these cases, media narratives tend to foreground individual responsibility while diverting attention from upstream determinants.

This pattern mirrors findings across public health, where media often emphasise individual choices over upstream determinants. As a result, migrant drownings are normalised as cultural or behavioural failings, while non‐migrant drownings are framed as situational or environmental misfortunes. Research emphasises that effective prevention requires culturally tailored and community‐led approaches [3], yet such strategies received minimal attention in reporting. Examples such as Swim Brothers and the Aqua English Project show that community‐led models strengthen participation and legitimacy. These cases illustrate that responsibilisation is not inevitable; shared responsibility becomes possible when migrants are positioned as partners rather than perpetual learners.

### 4.2 Visibility, Vulnerability and Prevention Messaging

Media narratives often make migrant drownings highly visible yet narrowly interpreted. Coverage frequently racialises risk, attributing vulnerability to cultural or linguistic identity rather than intersecting social determinants [30]. Similar deficit framings in maternal health reporting show how systemic drivers are downplayed, reinforcing what Frankenberg [31] and Hall [13] identify as the ‘unmarked’ positioning of whiteness: non‐migrant deaths are treated as accidents, while migrant deaths become racialised problems. These narrative patterns align with neoliberal discourses that shift responsibility from the state to individuals [32]. In migration contexts, this positions refugees and migrants as ‘deficit subjects’ who must assimilate, while structural barriers shaping risk remain obscured [33, 34, 35]. In drowning prevention, this logic appears in expectations that migrants must simply ‘learn to be Australian’, even when access to pools, culturally tailored programs, and affordable lessons is uneven. By contrast, drownings involving non‐migrants are more likely to be framed as situational misfortunes or environmental hazards.

Public health understandings of drowning emphasise the interaction of hazard, exposure and vulnerability [24]. Media focus on culture or behaviour disrupts this interplay, obscuring how socioeconomic position, geography and housing shape both exposure and the capacity to respond. Resilience also operates across individual, community and system levels, yet these dimensions rarely appear in reporting. This represents a missed opportunity to situate migrant drownings within broader ecologies of risk and resilience.

Visibility can still be valuable when major drowning events create ‘critical moments’ that draw inequities onto the policy agenda [36, 37]. Articles that include prevention advice, notably supervision, CPR and swimming between the flags, demonstrate how the media can contribute to public education [38]. However, visibility without agency risks depicting migrants as passive subjects of pity or fear [33]. The limited presence of migrant voices in Australian reporting heightens this concern. Greater inclusion of migrant perspectives could shift visibility from pity to partnership.

### 4.3 Climate as an Overlooked Driver of Inequity

There is increasing recognition that climate change may heighten drowning risk through rising temperatures, flooding and extreme weather [38, 39]. These risks are unevenly distributed. Migrants and low‐income communities are more often exposed while having fewer resources to adapt [2]. Yet climate rarely appeared in reporting on migrant drownings, even when it featured in coverage of non‐migrant cases. Instead, migrant drownings were framed through familiar narratives of cultural unfamiliarity or individual error.

This selective framing obscures the layered risks. Migrant communities often have disrupted access to water safety education (e.g., COVID‐19 pool closures) and are likely to be disproportionately affected by heatwaves, flooding and climate‐driven displacement [38]. Although drowning prevention organisations have begun to foreground climate risk, migrants are seldom consulted in adaptation planning. Disconnecting migrant drownings from climate discourse implies that migrants alone must adapt, rather than recognising how climate change compounds existing inequities.

### 4.4 Shifting From Deficit to Inclusionary Narratives

Despite persistent deficit frames, counter‐narratives of inclusion were evident. Coverage of Surf Life Saving's Nippers program regularly highlighted migrant families as active participants in aquatic culture, noting that more than 80 nationalities are involved (Sydney Morning Herald, 2 March, 2024). These stories positioned migrants as contributors to community safety and belonging, reflecting broader shifts within RLSSA and SLSA media campaigns towards more diverse representation.

Similar inclusionary framings occur in reporting on community sport, where multicultural participation is linked to social cohesion [40, 41]. This aligns with Hall's [13] argument that representation can move beyond ‘othering’ to construct shared identities. Such positive narratives provide valuable entry points for public health by framing migrant participation as a collective strength, not a deficit.

### 4.5 Media as a Platform for Equity‐Oriented Solutions

Media reporting also amplified expert calls for structural responses such as subsidised lessons, multilingual resources, and culturally tailored outreach. These reports recognise drowning prevention as a shared responsibility, not solely an individual burden. Reporting on community‐led initiatives and accessible programs helps normalise culturally responsive expectations across the sector.

This mirrors positive examples in other health domains, such as anti‐smoking campaigns and COVID‐19 public health messaging, where Australian media successfully disseminated multilingual and equity‐oriented information [27, 28, 29]. RLSSA and SLSA's annual campaigns further demonstrate how media partnerships can embed structural narratives across Australia. As seen in seatbelt promotion and Indigenous health advocacy, sustained reporting can shift public understanding towards systemic determinants [37, 42]. Positioning media as both critic and advocate enables drowning prevention to advance equity‐focused reform.

### 4.6 Engaging Migrant Communities as Partners

Although migrant voices were limited, when present they offered powerful testimony through vigils, fundraisers and cultural rituals. Such coverage demonstrates how migrants can be represented not only as ‘at risk’ but as catalysts for solidarity. Similar effects have been documented in cancer and mental health reporting, where migrant stories humanise systemic issues and generate empathy [43]. Evidence also shows the value of partnership: community‐led safety campaigns and co‐designed initiatives strengthen accuracy, legitimacy, and engagement. Willcox‐Pidgeon et al. [3] highlight the increasing involvement of migrant communities in water safety design, reinforcing the need to elevate these perspectives in public narratives.

### 4.7 Opportunities for Policy and Advocacy

Predictable spikes in media attention, particularly around youth, coastal and migrant drownings, create windows for evidence‐based intervention. These moments can be used to amplify culturally responsive campaigns, highlight structural reforms, and showcase migrant‐led initiatives. Seasonal, multilingual press kits could align prevention efforts with peak news cycles, while emphasising migrant participation in programs like Nippers could counter persistent deficit framings. These opportunities align with the Australian Water Safety Strategy 2030's focus on equity, inclusion, and accessibility [24]. As with other ‘critical moments’ in youth suicide or Indigenous health reporting [42], media attention can help open policy windows for reform when used strategically.

### 4.8 Points for Action

These findings suggest several practical points for action. First, journalists and editors can strengthen equity‐oriented reporting by routinely including structural context (access to lessons, patrol coverage, affordability, multilingual resources) alongside behavioural messaging and by broadening the range of quoted voices beyond official agencies to include community leaders and affected families where appropriate. Second, water safety organisations can support this shift by providing season‐ready media packs that include culturally responsive messaging, spokesperson lists that include multicultural/community partners, and case examples that highlight participation pathways rather than deficit narratives. Third, policy and program design can use predictable media ‘spikes’ (e.g., peak summer reporting) as windows to amplify structural prevention measures (subsidised lessons, improved pool access, multilingual signage, and community‐led programs), so that prevention does not rely on individual adaptation alone. Finally, future research should extend beyond newspapers to broadcast, social media and multilingual outlets, and incorporate migrant perspectives directly to test whether media framing influences risk perception, trust, and program uptake.

## 5 Strengths and Limitations

Strengths of this study include the use of a transparent CDA framework alongside inductive thematic analysis, and the comparative design examining migrant and non‐migrant reporting across high‐readership mastheads. Limitations include reliance on ProQuest‐indexed newspaper articles, which exclude broadcast media, social media, and community‐language outlets that may frame migrant drownings differently. The sample is also shaped by ownership concentration and syndication, which may reduce diversity of frames. Although keyword frequency supported comparison, interpretation remained qualitative and context‐dependent. We did not include audience reception data; therefore, implications for public attitudes are inferential and should be tested in future work.

## 6 Conclusion

Australian media coverage of drowning is both a site of racialised deficit framing and a potential partner in advancing equity. Migrant drowning stories, while often positioned through cultural unfamiliarity, are highly visible and emotionally resonant, creating openings for prevention and community solidarity. Inclusionary narratives, especially those centred on family participation and community programs, demonstrate that alternative stories are possible and persuasive.

By acknowledging the diversity of migrant experiences and amplifying migrant voices, journalists, policymakers and advocates can help shift reporting towards structural and participatory framings. This aligns with wider media scholarship showing that positive reframing is achievable when timely, culturally informed stories are made available. The task ahead is not only to critique racialised narratives but to work with media and sector partners to embed equity, inclusion and migrant participation at the core of drowning prevention efforts.

## Funding

This research was supported by an Australian Government Research Training Program (RTP) Scholarship and the National Health and Medical Research Council (NHMRC) (Grant RG244093).

## Ethics Statement

This study analysed publicly available newspaper articles retrieved via the ProQuest database and did not involve recruitment of human participants, collection of identifiable private information, or interaction with individuals. As such, formal Human Research Ethics Committee approval and participant consent were not required for this document‐based analysis.

## Conflicts of Interest

The authors declare no conflicts of interest.

## Data Availability

The data that support the findings of this study are available on request from the corresponding author. The data are not publicly available due to privacy or ethical restrictions.
